# Hydrogen Embrittlement Evaluation of Micro Alloyed Steels by Means of *J*-Integral Curve

**DOI:** 10.3390/ma12111843

**Published:** 2019-06-06

**Authors:** Marina Cabrini, Ennio Sinigaglia, Carlo Spinelli, Marco Tarenzi, Cristian Testa, Fabio Maria Bolzoni

**Affiliations:** 1 Department of Engineering and Applied Sciences, University of Bergamo, 24044 Dalmine (BG), Italy; cristian.testa@unibg.it; 2 INSTM Unità di Ricerca Bergamo, 24044 Dalmine (BG), Italy; 3 CSGI Unità di Ricerca Bergamo, 24044 Dalmine (BG), Italy; 4 Transitgas AG Zurich (Ch), 8001 Zurich, Switzerland; sinigaglia@transitgas.ch; 5 Eni S.p.A TECM, 20097 San Donato Milanese (MI), Italy; carlo.maria.spinelli@eni.com; 6 Snam S.p.A. DIREZIONE TECNICA, 20097 San Donato Milanese (MI), Italy; marco.tarenzi@snam.it; 7 Dipartimenti di Chimica, Materiali e Ingegneria Chimica G. Natta Politecnico di Milano, 20131 Milano (MI), Italy; fabio.bolzoni@polimi.it

**Keywords:** hydrogen embrittlement, pipeline steel, mechanical fracture, cathodic protection

## Abstract

The aim of this work is the evaluation of the hydrogen effect on the *J*-integral parameter. It is well-known that the micro alloyed steels are affected by Hydrogen Embrittlement phenomena only when they are subjected at the same time to plastic deformation and hydrogen evolution at their surface. Previous works have pointed out the absence of Hydrogen Embrittlement effects on pipeline steels cathodically protected under static load conditions. On the contrary, in slow strain rate tests it is possible to observe the effect of the imposed potential and the strain rate on the hydrogen embrittlement steel behavior only after the necking of the specimens. *J* vs. Δa curves were measured on different pipeline steels in air and in aerated NaCl 3.5 g/L solution at free corrosion potential or under cathodic polarization at −1.05 and −2 V vs. SCE. The area under the *J* vs. Δa curves and the maximum crack propagation rate were taken into account. These parameters were compared with the ratio between the reduction of area in environment and in air obtained by slow strain rate test in the same environmental conditions and used to rank the different steels.

## 1. Introduction

Environmental Assisted Cracking (EAC) phenomena take place when a specific aggressive environment and a mechanical load over a critical threshold value act simultaneously on a susceptible material. Different mechanisms have been proposed for EAC phenomena, and one of these is Hydrogen Embrittlement (HE), which takes place when atomic hydrogen enters into the metal lattice. At room temperature, adsorption of atomic hydrogen on a metal surface can be caused by the electrochemical reduction of water during steel corrosion or by the application of a cathodic potential (CP) that is lower than the thermodynamic threshold for hydrogen evolution. Buried pipelines and sea lines are normally protected against general corrosion by means of CP. Although the transport of hydrocarbons through pipelines is an established and secure technology, several cases of EAC resulting from the effect of hydrogen on steel have been reported globally [1,2]. In the presence of altered microstructures, i.e., hard spots, hydrogen can favor the initiation and propagation of brittle fracture [3,4]. Hard spots in pipeline steels may be caused by the manufacturing process or during the execution of welding, mainly on steel with high content of carbon, sulfur and manganese, and are characterized by a hard, untempered martensite and bainite microstructure. The critical role of the hard spots can be ascribed to the presence of untempered martensite, which is the microstructure most susceptible to HE [5,6].

Furthermore, Razzini et al.—by visualizing the hydrogen distribution in an artificial hard spot on API 5L X60 steel by using photo-electrochemical technique—demonstrated that the solubility of hydrogen in the heat affected zones of hard spots is higher than the base material [7]. In the absence of microstructural alterations, pipeline steels are immune to HE under cathodic protection, even at very low cathodic potentials [8]. The nominal desired potential range for a pipeline under cathodic protection is between −850 and −1200 mV, measured with respect to a Cu/CuSO_4_ reference electrode, but more negative potentials can be present in the correspondence of the remote anodes, especially in the case of high soil resistivity [9]. Only a few cases have been observed on buried pipeline under slow plastic deformations [10], mechanical damage and landslides [11].

Recently, particular attention has been given to the HE risks, especially taking into account the possibility of increasing the mechanical properties of pipeline steel [12]. In fact, it is well known that the susceptibility to HE increases with the increase in the tensile resistance of the steel [13]; on the other hand, increasing the steel grade is the most convenient option for long-distance and high-pressure pipelines [14]. However, the behavior of pipeline steels with respect to HE phenomena is currently not well defined.

The International Standards on CP have been altered to introduce critical values of negative potentials that cannot be exceeded in the case where high strength steels are used. For example, the ISO 15589-1:2015 (Petroleum, petrochemical and natural gas industries—Cathodic protection of pipeline systems—Part 1: On-land pipelines) and the ISO 3183 standard specify that the cathodic protection potential of high strength steels (yield strength above 550 MPa) shall be determined correctly in order to avoid the risk of hydrogen formation at the metal surface. Unfortunately, neither of the above-mentioned standards suggest experimental procedures for determining the immunity/susceptibility to HE.

Many laboratory studies have been carried out on pipeline steels [1,10,13,15,16,17,18,19,20,21,22,23,24,25,26]; nevertheless, some aspects of this phenomenon remain poorly understood.

Laboratory tests have also confirmed the immunity of pipeline steels to HE with applied cathodic potentials lower than −2 V vs. SCE under static load conditions (constant load or constant deformation) [27,28]. HE phenomena in this class of steels require the presence of dynamic straining in the plastic field. Several theories have underlined the role of hydrogen in the plastic strain of steels, including the decrease in cohesive force resulting in brittle fracture (Hydrogen Enhanced Decohesion—HEDE) [29,30] and the Hydrogen Enhanced Localized Plasticity theory—HELP [31], as well as the Hydrogen Enhanced Strain-Induced Vacancies theory—HESIV [32], which tries to explain the effect of hydrogen on ductile fracture. Srinivasan and Neeraj suggested failure mode transitions from ductile to quasi-brittle fracture with increasing yield strength and/or triaxiality of the stress state. The coexistence and applicability of different theories (HELP, HEDE and HESIV) seems to be necessary to explain this mechanism [33]. The Adsorption-Induced Dislocation Emission (AIDE) mechanism is based on hydrogen-induced weakening of interatomic bonds (as in HEDE), but with crack growth occurring by localized slip (as in HELP) [34].

The Slow Strain Rate (SSR) test is one of the most used techniques for evaluating the EAC susceptibility of metals in determinate environments [15], but on cathodically protected pipeline steels, the environmental effects only become evident after necking [35]. Furthermore, secondary cracks are only observed in the necking zones [10]. Slow bending tests have been developed in order to provide an effective alternative test to SSR for assessing hydrogen embrittlement resistance of steels with tensile yield strength (TYS) ranging between 420 and 770 MPa [36] and to evaluate the effect of plastic deformation [37].

Fracture mechanics are also often used to study the hydrogen effect on crack propagation [25,38], but the high toughness of pipeline steel makes it very difficult to obtain true plain stress conditions. Li et al. [39] studied hydrogen embrittlement in single edge notch tension (SENT) testing of high-strength pipeline steel using specimens with various thicknesses. The specimens were pre-charged with hydrogen and then tested in air. The results showed a hydrogen effect only when the thickness of the specimen was increased due to the constrained crack tip deformation in the hydrogenated specimens. On the other hand, it is well known that the hydrogen embrittlement phenomena are enhanced when the hydrogen evolution is simultaneous with plastic strain, and the hydrogen supply at the crack tip depends on the loading mode and strain rate [40,41,42,43,44,45,46,47,48,49,50,51,52,53,54,55].

Linear elastic mechanical fracture cannot be used in dynamic continuous deformation, like the conditions needed to have HE in pipeline steels, because pipeline steels have a good toughness and a very high ductile behavior also when they are fully saturated with atomic hydrogen. The effect of hydrogen, on the other hand, can also be evidenced in terms of ductile crack initiation and rate of crack growth in low-alloyed steels [32]. The study of ductile or mixed brittle/ductile fracture requires an elastic-plastic mechanical fracture approach to establish the HE susceptibility of the tested steels.

The ductile fracture of metals, in which plastic deformation dominates at the crack tip and the material resistance against fracture increases as the crack grows, is often described in a resistance curve format using the *J*-integral vs. the crack’s growth Δa [56]. The *J*-integral is the strain energy release rate for a crack in a body subjected to monotonic loading, under quasi-static conditions, both for the linear elastic materials and for materials that experience small-scale yielding at the crack [57].

In this paper, the *J*-integral vs. Δa curves were obtained for different grade steels in air and in NaCl 3.5 g/L solution at the free corrosion potential and cathodically polarized at −1.05 and −2 V vs. SCE using the potential drop method to evaluate the crack growth [58,59]. The crack growth rate and the area under the *J* vs. Δa curve were considered to evaluate the environmental effects, in comparison with the standardized slow strain rate tests results. These parameters were then used to rank the hydrogen susceptibility of the different tested steels.

## 2. Materials and Methods

**Figure 1:**
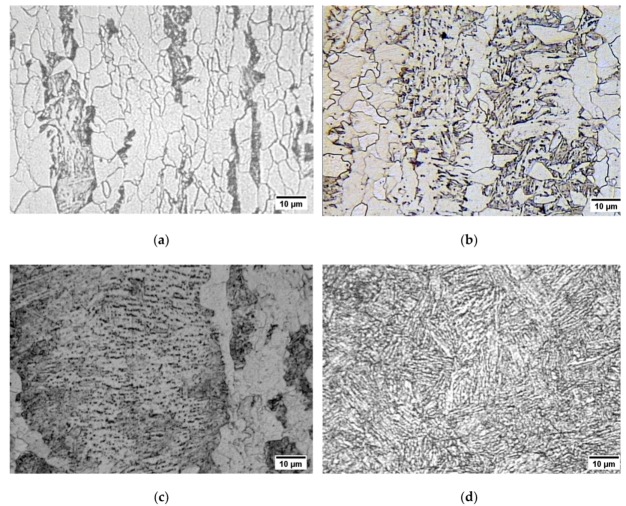
Microstructure of the testes (500×): (**a**) X70SS steel (Nital attack); (**b**) X80 steel (Picral attack); (**c**) X100 steel (ferric chloride attack); (**d**) 30NiCrMo12 steel (Nital attack).

The chemical compositions of the tested steels are reported in Table 1. The X70SS steel is an API (American Petroleum Institute) 5L Thermo-Mechanically Controlled Processed ferritic-pearlitic steel for sour service; it shows grains oriented along the hot rolling direction (Figure 1a). The “sour” grade warrants a low MnS inclusions tenor. The steels referred to as X80 and X100 are proposed pipeline steels sourced from an experimental melting, obtained by controlled rolling and accelerated cooling. The X80 has a bainitic microstructure, with ferrite islands (Figure 1b), while the X100 steel has a martensitic and bainitic microstructure (Figure 1c). The 30NiCrMo12 is a quenched and tempered steel with a tempered martensitic structure (Figure 1d). The mechanical properties of the steels are summarized in Table 2.

All tests were carried out in air (for comparison) and in aerated 3.5% NaCl (Carlo Erba RPS analytical grade reagents, Cornaredo Italy) aqueous solution, at free corrosion potential and potentiostatically polarizing the specimens at −1 or −2 V vs. SCE with an Amel mod. 352 (Italy) potentiostat/galvanostat and graphite as counter. All tests were carried out at 23 ± 1 °C.

**Figure 2:**
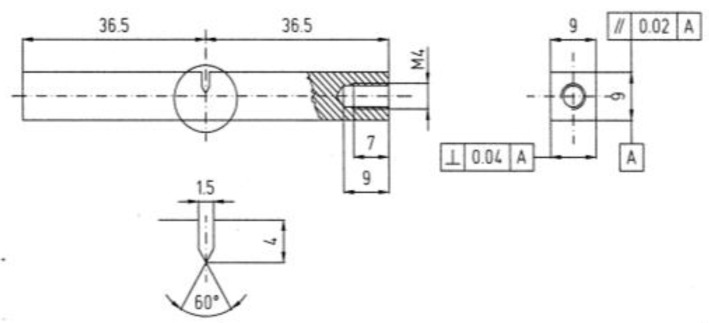
SENB tests specimen.

The experimental determination of the crack resistance J versus crack length a (J-R curves) were conducted on Single Edge Notch Bend (SENB) (Figure 2) with the potential drop (PD) method described in [60]. Before the bending tests, the specimens were pre-cracked to represent a mechanical notch using an electromagnetic machine with a frequency of 220 Hz and an R ratio of 0.1 until the initial crack length equaled 50% of the specimen width. The PD method is based on the change in electrical resistance of a specimen with crack length, independently of the type of loading. The change in electrical resistance results in a change of the potential drop between two measuring points across the crack. A DC Potential Drop Reverse Current Crack Size Monitoring Units has been “in house” engineered to supply the right current, stability necessary for the Cycling Reverse DC potential drop method and it has been adapted with a voltage measurement to permit synchronized data storage. The power DC power supply unit provides a direct current of 15 amps within a voltage rating of 8 volts. It was based on solid-state polarity-reversing units; the adoption of this DC polarity inversion was chosen to supply constant current and to enlarge the voltage drop magnitude; furthermore, the DC power supply is less stressed, as the current output remains constant wherever with minimal switching transients. The temperature drift was evaluated to be within 0.05%/°C. The data recording and managing software was developed internally and was based on an engineering platform from the LabView (National Instrument) platform. The measuring units were equipped with two channels for active and reference probes. The “as it is signals” were firstly amplified by differential units and then filtered with the aim of providing a maximum gain of 20k.

Polarization inversion was adopted to avoid the effect of the solution and temperature and to reduce the electric noise; two specimens, one loaded and one un-loaded, were connected in series and DC polarized at 8 V using polarization inversion; in this configuration, in fact, the potential values obtained by polarity opposite are subtracted and appropriately normalized.

A dimensionless expression is obtained:

(1) $$U=\frac{U_{i}^{+}-U_{i}^{-}}{U_{r}^{+}-U_{r}^{-}}$$

where *U* is a dimensionless ratio that expresses the extent of the growth of the crack for a specific set composed of the test specimen and of the relative reference specimens, to avoid the effects due to fluctuations of the electrical, temperature, and local polarization parameters; *U*^+^ and *U*^−^ represent the values of ohmic drop measured during the two moments in which the current is made to flow in a sense and in the inverse sense in order to deduct the effects of local polarization during the permanence of the circulation of current in a verse; the subscript *i* is related to the specimen subjected to fracture mechanic test and *r* refers to the reference specimen immersed in the same solution but not subjected to mechanical testing. To guarantee a constant value of the reference potential *Ur*, this measurement was carried out on the second specimen of the same materials, not subject to deformation, placed in the same environmental conditions, electrical connected in series and placed close to the stressed specimen to avoid ohmic drop in the metallic conductor and erase the effects of possible variations of temperatures. The difference in potential among the specimens were measured using a microvoltmeter with high accuracy (0.1 µV) and high stability (drift 0.02 µV/°C). Tests were conducted by displacement control, at 0.001 m/s. During the bending tests, the value of the displacement, load, time and potential drop were measured every 300 s. Two repetitions for each condition were executed.

The value of *a* by the potential drop can be evaluated by the Johnson’s equation [61,62]:

(2) $$a=\frac{2W}{\pi }cos^{-1}\frac{cosh\left(\pi y/2W\right)}{cosh\left\{\left(U/U_{0}\right)cosh^{-1}\left[cosh\left(\pi y/2W\right)/cos\left(\pi a_{0}/2W\right)\right]\right\}}$$

where *a* is the crack length corresponding to potential drop *U*, *y* is one half of the potential gauge span (4.5 mm), *W* is the specimen width, and *a*_0_ and *U*_0_ are the initial crack length and the initial potential drop measurement, respectively.

From the crack lengths the *J*-integral can be calculated on the basis of the ASTM 1820-18 by following equation:

(3) $$J_{i}=J_{ei}+J_{pli}$$

*J_e_* is the elastic component, evaluable from the following equation:

(4) $$J_{ei}=\frac{K_{i}^{2}\left(1-\nu^{2}\right)}{E}$$

where *K_i_* is the stress intensity at the *i*-th data point, *ν* is Poisson’s ratio, *E* is the Young’s modulus of the material; *K_i_* is given from the equation:

(5) $$K_{i}=\frac{P_{i}S}{{\left(BB_{N}\right)}^{\frac{1}{2}}W^{\frac{3}{2}}}\times \frac{3{\left(\frac{a_{i}}{W}\right)}^{\frac{1}{2}}\left[1.99-\left(\frac{a_{i}}{W}\right)\left(1-\frac{a_{i}}{W}\right)\left(2.15-3.93\left(\frac{a_{i}}{W}\right)+2.7{\left(\frac{a_{i}}{W}\right)}^{2}\right)\right]}{2\left(1+2\frac{a_{i}}{W}\right){\left(1-\frac{a_{i}}{W}\right)}^{\frac{3}{2}}}$$

where *P_i_* is the load at the *i*-th data point, *B* is the specimen thickness, *B_N_* is the specimen net thickness, *a_i_* is the crack size at the *i*-th data point, and *S* is the span dimension.

*J_pli_* is the plastic component of *J_i_* at the *i*-th data point, and is evaluated by means of the relationship:

(6) $$J_{pli}=\left[J_{pli-1}+\frac{\eta_{pli-1}}{b_{i-1}}\times \frac{A_{pli}-A_{pli-1}}{B_{N}}\right]\left[1-\gamma_{pli-1}\frac{a_{i}-a_{i-1}}{b_{i-1}}\right]$$

where *J_pli−1_* is the plastic part of *J*-integral for the (*i*−1)-th data point and assuming the initial plastic component *J_pl0_*, equals to zero; *b_i-1_* is the uncracked ligament for the (*i*−1)-th data point and is equal to *W-a_i−1_*; *η_pli−1_* is a dimensionless parameter that relates plastic work done on a specimen to crack growth resistance, and is 1.9 for SENB specimens [62,63]; and *γ_i−1_* is a function to correct the *J*-integral evaluated by the *η_pli−1_* parameter in the crack growth situation [64] and is equal to 0.9 for SENB specimens. The quantity *A_pli_−A_pli−1_* is the increment of the plastic area under the load versus the plastic load-line displacement record between lines of constant plastic displacement *v_pli_* and *v_pli−1_* and can be calculated from the following equation:

(7) $$A_{pli}=A_{pli-1}+\frac{\left(P_{i}+P_{i-1}\right)\left(\nu_{pli}-\nu_{pli-1}\right)}{2}$$

where *v_pli_* is the plastic part of the *i*-th load-line displacement data point:

(8) $$v_{pli}=v_{i}-P_{i}\left\{\frac{1}{EB_{e}}{\left(\frac{S}{W-a_{i}}\right)}^{2}\times \left[1193-1.98\left(\frac{a_{i}}{W}\right)+4.478{\left(\frac{a_{i}}{W}\right)}^{2}-4.443{\left(\frac{a_{i}}{W}\right)}^{3}+1.739{\left(\frac{a_{i}}{W}\right)}^{24}\right]\right\}$$

where *v_i_* is the load-line displacement at the *i*-th data point and *B_e_* is the effective thickness:

(9) $$B_{e}=B-\frac{{\left(B-B_{N}\right)}^{2}}{B}$$

**Figure 3:**
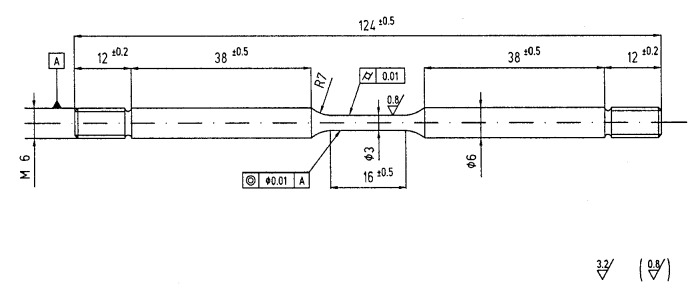
SSR tests specimens.

Slow strain rate tests were performed on cylindrical tensile specimens (Figure 3) obtained according to the ISO 7539-7 (2005) standard. The specimens were obtained near the external surface of the original pipe, at minimum depth. Before the tests, the specimens were finished with abrasive paper (1200 grit) to eliminate the strain-hardened layer induced by mechanical working. Finally, they were degreased with acetone (Carlo Erba RPA reagents, Cornaredo Italy). The SSR tests were carried out through a tensile machine, with four independent stations of loading and displacement rate adjustable between 5·10^−7^ and 5·10^−3^ mm·s^−1^. A strain rate of 10^−6^ s^−1^ was adopted. During the test, the load was monitored as a function of time in order to estimate stress vs. strain curves. After the tests, the total elongation (A) and the reduction of area (Z) were measured on the specimens. The tests were conducted twice.

The susceptibility to environmental cracking was evaluated through the hydrogen embrittlement index, evaluated on the basis of the reduction in area ratio between the test in the environment and in air, following the relationships:

(10) $$HE_{index\;Z}=\left(1-\frac{Z_{environment}}{Z_{air}}\right)\times 100$$

## 3. Results and Discussion

**Figure 4:**
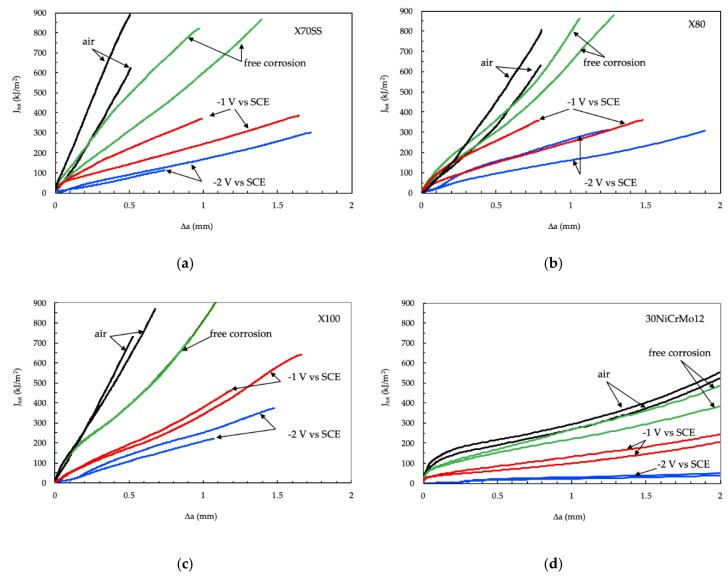
Results of *J*-integral tests. (**a**) X70SS steel; (**b**) X80 steel; (**c**) X100 steel; (**d**) 30NiCrMo12 steel.

**Figure 5:**
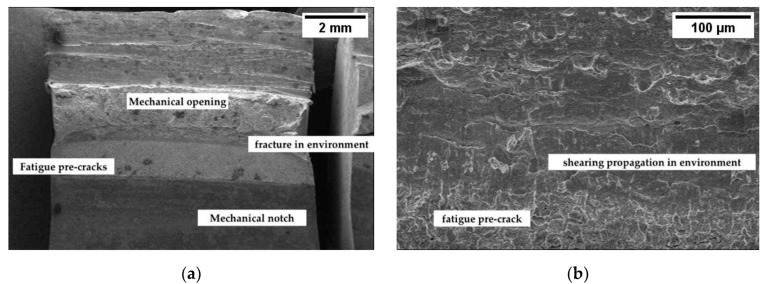
Fracture surface in *J*-integral test for X100 steel at free corrosion potential: (**a**) macro and (**b**) close-up view of the crack propagation zone.

Figure 4 reports the *J_tot_* vs. Δa curves obtained for the different steels. The *J_tot_* vs. Δa curves in air are similar for the three pipeline steels (X70SS, X80 and X100), regardless of their different mechanical properties; on the contrary, the martensitic steel shows *J_tot_* values lower than those of the other steels at the same crack extension, indicating a decrease in toughness. When the tests were conducted in NaCl 3.5 g/L solution at the free corrosion potential, the *J_tot_* values decreased for all the steels at every Δa, indicating a decrease in energy for the propagation of the fracture. In all cases, no brittle zones were present on the fracture surface, only on the shearing area (Figure 5).

**Figure 6:**
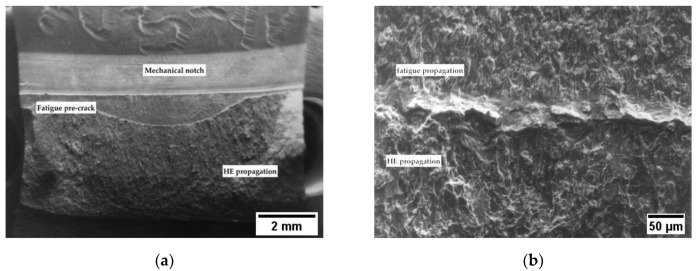
Fracture surface in *J*-integral test for X70 steel polarized at −1 V vs. SCE: (**a**) macro and (**b**) close-up view of the fatigue pre-cracking crack propagation transition zone.

**Figure 7:**
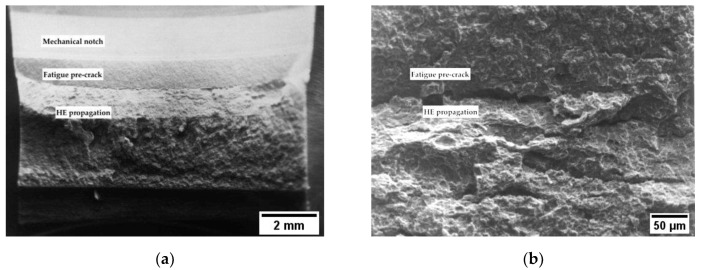
Fracture surface in *J*-integral test for X80 steel polarized at −1 V vs. SCE: (**a**) macro and (**b**) close-up view of the fatigue pre-cracking crack propagation transition zone.

**Figure 8:**
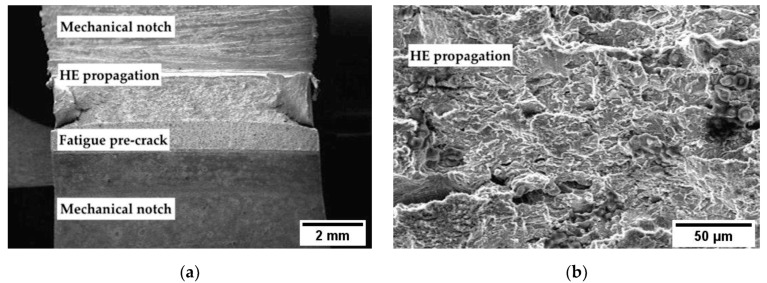
Fracture surface in *J*-integral test for X100 steel polarized at −1 V vs. SCE: (**a**) macro and (**b**) close-up view of the crack propagation zone.

Applying the cathodic potentials, the *J_tot_* dramatically decreases, indicating a meaningful effect of hydrogen in fracture propagation. The decrease in the *J_tot_* vs. Δa grows more pronounced as the cathodic polarization increases, and corresponds to the presence of brittle areas on the fracture surface (Figure 6, Figure 7 and Figure 8).

**Figure 9:**
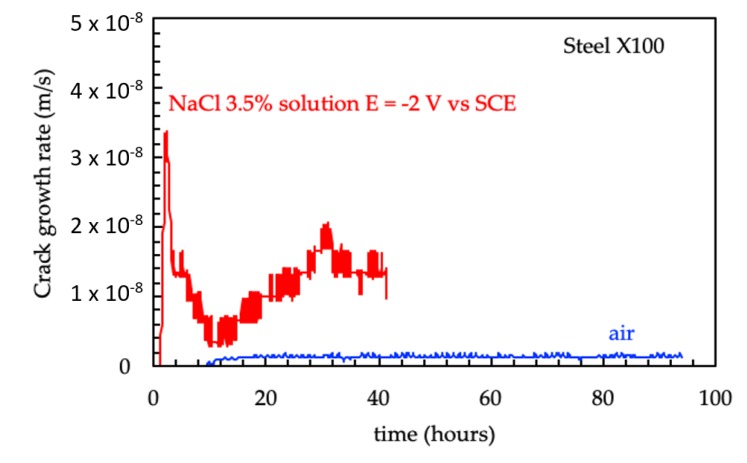
Crack growth rate as a function of time for the API 5L X00 steel in air (blue curve) and in NaCl 3.5% solution at −2 V vs. SCE (red curve).

At the end of the tests, the crack propagation rate $\left(v=\frac{da}{dt}\right)$ was calculated by cubic spline approximation of experimental data [65]. An example of the crack growth rate of the API 5L X100 steel obtained in air and in NaCl solution, with cathodic polarization of −2 V vs. SCE, is presented in Figure 9. The crack of the cathodically protected specimen begins to propagate at loads lower than that of the samples in air; moreover, its speed is noticeably superior. The crack growth rate in air is also practically constant throughout the test time, lower than 2·10^−9^ m/s, while in the environment, it peaks in the initial moments, and decreases over the following ten hours, before increasing again in the hours after that to settle in a value range of 5·10^−8^ m/s.

**Figure 10:**
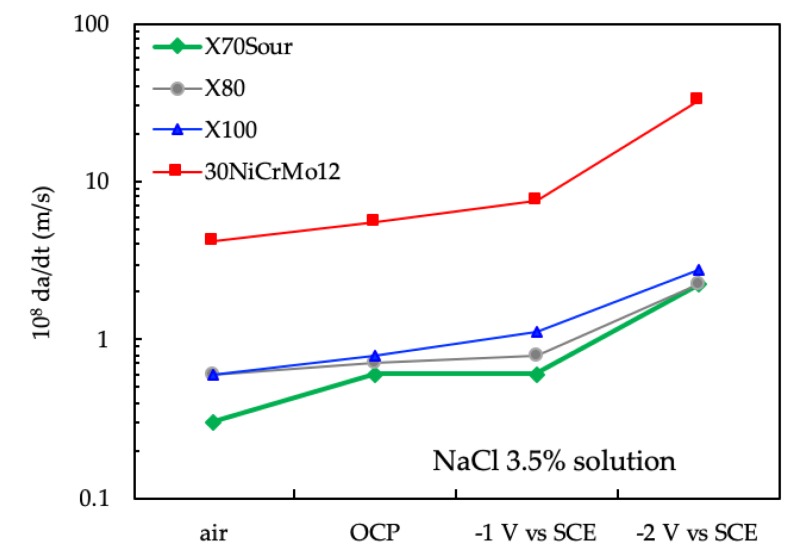
Effect of the environment on the maximum crack growth rate.

The peak values of the crack growth obtained in NaCl 3.5% solution were higher than those obtained in air, and increase with the increasing of cathodic polarization of the specimens, as shown in Figure 10. The crack growth rate in air increases with the increasing of the tensile resistance of the steels. The maximum crack growth rate of 30NiCrMo12 steel was markedly higher than that of the three pipeline steels. At the same time, in NaCl 3.5% solution, the crack growth rate was higher than in air, and further increases were observed when the specimens were cathodically protected.

From the *J* vs. Δa curves, a parameter for quantifying the effect of hydrogen on the fracture propagation was obtained. With reference to the ASTM 1820-18 standard, the blunting line, which describes the initial behavior of the crack before ductile growth, was drawn with the equation:

(11) $$J=2\sigma_{Y}\Delta a$$

The region of validity of the data is between two lines parallel to the blunting line at a distance of 0.15 and 1.5 mm (exclusion line). The first line represents the lower limit, beyond which the growth of the crack is considered stable, the second limit the condition under which the *J*-integral is fulfilled.

To obtain an estimation of the energy needed for crack growth, the area under the *J* vs. Δa curve (*S*) was calculated using a polynomial regression of the curves *J* vs. Δa, until R > 99.9% was achieved. The polynomial curve was then integrated between 0.2 and 0.5 mm. The value 0.5 mm was chosen by taking into account the maximum growth observed in the test in air.

**Figure 11:**
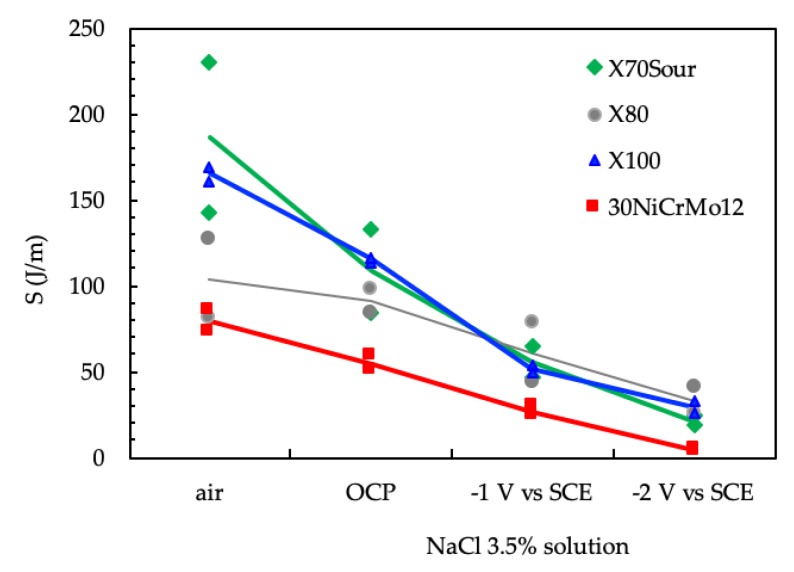
Effect of the environment on the area under the curve *J* vs. Δa for the tested steel.

The results are summarized in Figure 11. The area under the curves obtained in air tends to decrease with increasing tensile resistance in the steel, and, for each steel, this value markedly decreased in the presence of the environment and with the application of the cathodic polarization. For all steels, the energy for the crack growth decreased, passing to OCP to cathodic polarization (−1 V vs. SCE), and reached a minimum level in the presence of high cathodic polarization, i.e., −2 V vs. SCE. These conditions correspond to high surface hydrogen concentration. In the presence of high polarization, the differences between the steels are strongly reduced.

The ratios between the values in the environment and those in air were calculated in order to obtain an index for the hydrogen effect, following the relationship:

(12) $$HE_{index\;SJ}=\left(1-\frac{S_{environment}}{S_{air}}\right)\times 100$$

This index was compared with the results of the SSR tests.

**Figure 12:**
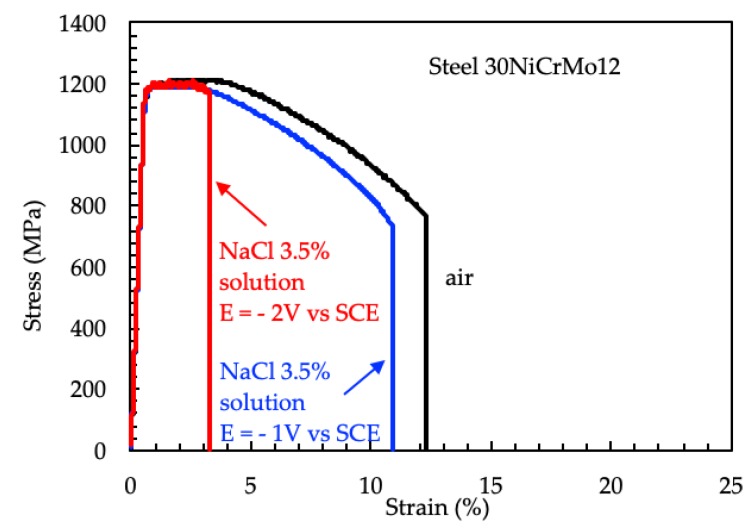
Stress vs. strain curves of the 30NiCrMo12 steel in air and in NaCl 3.5% solution with cathodic protection.

The stress vs. strain curves obtained in NaCl 3.5% solution with cathodic protection are significantly different from those obtained in air only for very high polarization (Figure 12).

**Figure 13:**
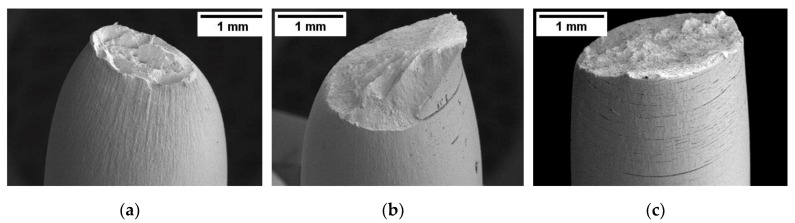
SEM images of the specimens of the X100 steel after the SSR test in NaCl 3.5% (strain rate 10^−6^ s^−1^) (**a**) at the open circuit potential; (**b**) polarized at −1 V vs. SCE; (**c**) polarized at −2 V vs. SCE.

The analysis of the fracture surface evidenced an absence of the hydrogen embrittlement effect at the OCP (Open Circuit Potential) (Figure 13a) and the presence of brittle areas and secondary cracks on the specimens polarized at −1 V vs. SCE; in all cases, the hydrogen effect took place only after the insurgence of the necking, under strong tri-axial stress conditions (Figure 13b).

Finally, the presence of quasi cleavage areas was observed on nearly all of the fracture surfaces on specimens polarized at −2 V vs. SCE (Figure 13c).

**Figure 14:**
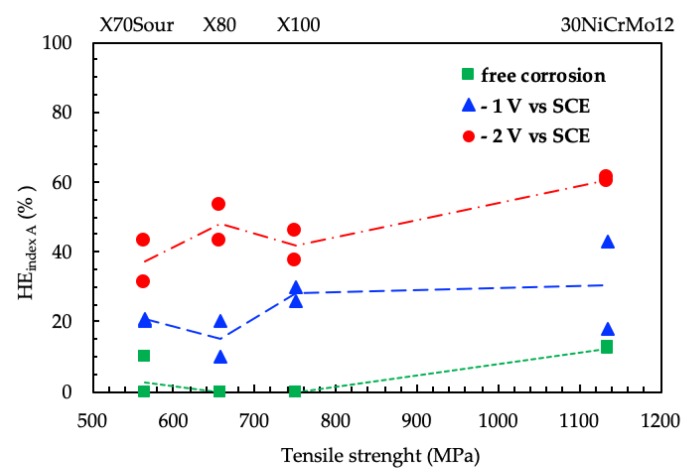
Effect of the mechanical properties of the steels on the *HE_index_* in SSR tests.

Previous works have demonstrated that the value of the reduction of area is the most sensible parameter for assessing hydrogen embrittlement phenomena in this class of steel under cathodic polarization [8,28]. Figure 14 shows the HE indexes relative to the reduction of area as a function of the Ultimate Tensile Strength of the steels.

For all steels, the *HE_indexZ_* at the free corrosion potential is zero, or very low in the case of 30NiCrMo12, indicating the practical absence of hydrogen embrittlement effect. On the contrary, a marked increasing of the *HE_indexZ_* was observed under cathodic polarization. The HE effects increase with the increasing of the cathodic polarization. The HE indexes slightly increase with the mechanical properties of the steels, especially for the lowest polarization potential (−2 V vs. SCE).

**Figure 15:**
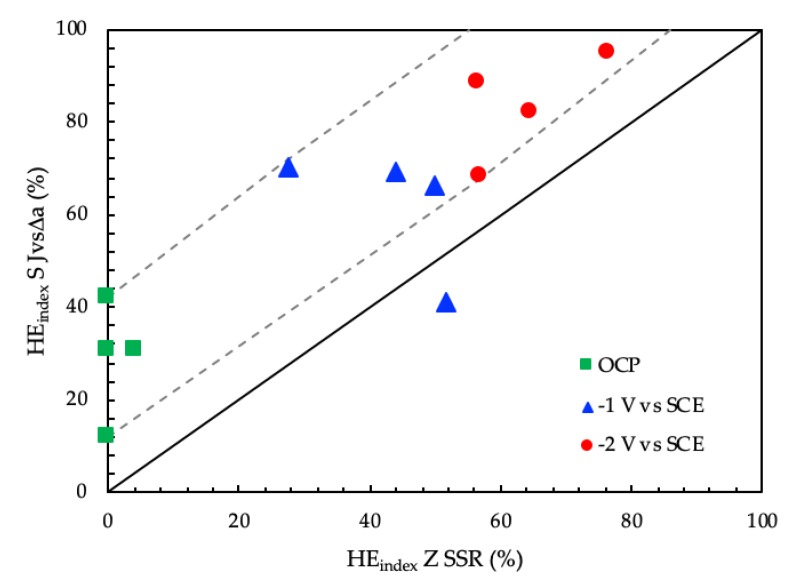
Correlation between the average values of the *HE_index SJ_* vs. *HE_index Z SSR._*

Comparing the two hydrogen embrittlement indexes (*HE_index SJ_* vs. *HE_index Z SSR_*) (Figure 15), the *J*-integral tests seem to be more able than SSR to indicate the hydrogen effect inside the metal; in fact, the *HE_index SJ_* index is generally above the straight at 45°. In addition, by means of the *J*-integral, it is possible to demonstrate the influence of the environment and the steel microstructure and mechanical properties on the fracture propagation at free corrosion potential.

**Figure 16:**
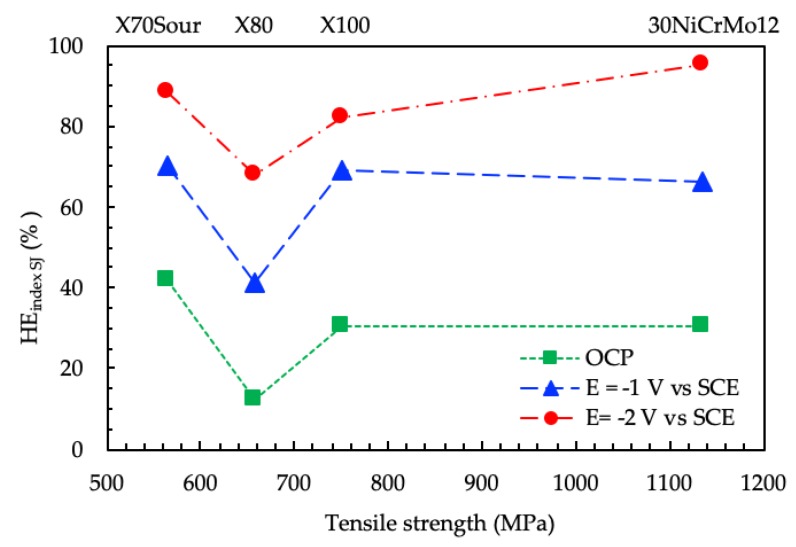
Effect of mechanical properties on the *HE_indexJS._*

The free corrosion potential of carbon steel in aerated NaCl 3.5% solution is about −0.65 V vs. SCE, which is practically the same value of the thermodynamic potential for hydrogen evolution at pH 7. However, as discussed in more detail in [28], the reduction of oxygen that takes place in aerated solution on metal surface increases the local pH; the alkalization is estimated to increase the pH of the metal surface to approximately pH 11. At this pH, the hydrogen equilibrium potential decreases, inhibiting the hydrogen evolution and, as a consequence, the hydrogen embrittlement. In his mechanochemistry theory, Gutman [66] demonstrated that mechanical deformation enhances steel corrosion. Xu and Cheng [67,68] studied the corrosion of pipeline steels under deformation and concluded that the mechano-electrochemical effect developed by the steel was limited to the elastic region, but became significant under plastic strain [69]. High plastic straining conditions were present at the crack tip of the SENB specimens used in the *J*-integral tests, which had been fatigue pre-cracked. Strain-assisted dissolution with initiation of pits in aerated NaCl 3.5% solution at free corrosion potential was observed in the first steps of the initiation of corrosion-fatigue cracks [70]. Strain-assisted dissolution can reduce the energy required for crack growth. The growth of the pit depends on the steel microstructure, i.e., the presence of inclusions and the orientation of the grain boundaries. This could explain the higher embrittlement index of the X70SS steels, which have larger grain size oriented perpendicularly to the crack propagation direction. The X80 steel, which has a very small grain size, showed the lowest value of embrittlement index, while the martensite or bainite phase present in the X100 steel showed a behavior similar to that of the fully martensitic 30NiCrMo12 steel (Figure 16).

In the presence of hydrogen, the crack growth morphology changes from ductile to brittle, and the *HE_indexJS_* increases. The embrittlement effect is not only related to the mechanical properties, but also depends on the microstructure. Ferrite/acicular microstructure has higher resistance to stress corrosion cracking in acidic environments than ferrite/pearlite or ferrite/bainite microstructures [71]. Many authors have reported better stress corrosion cracking (SCC) and hydrogen-induced cracking (HIC) resistance in acicular ferrite than in polygonal ferrite and pearlite [72,73,74]. A similar behavior can also be assumed under cathodic protection [28]. The presence of hard constituents of untempered martensite decreases the cracking resistance [57,73,74,75]. The fine bainitic and acicular ferrite microstructure of the X80 steel gives it higher hydrogen embrittlement resistance than the lower grade X70SS, characterized by a polygonal ferritic microstructure, and also better HE resistance than the higher grades martensitic steels. The X100 steel did not show any appreciable difference with respect to the X70SS steels, characterized by lower tensile strength, while a worsening was evident for 30NiCrMo12 steel only at the lower potential considered (−2 V vs. SCE).

## 4. Conclusions

From the results illustrated above, the following conclusions can be drawn:

- The *J*-integral vs. Δa determination is a suitable technique to characterize pipeline steels, which are not susceptible to hydrogen embrittlement under static loading conditions, but can exhibit HE susceptibility under almost-static loading conditions, such as in Slow Strain Rate tests.
- The ratio of the areas under the *J*-Δa curve in the environment and in air was chosen as a parameter for characterizing the hydrogen embrittlement of the different steels.
- The X80 steel showed the highest hydrogen embrittlement resistance, while X70SS, X100 and 30CrNiMo had similar behaviors.

## Figures and Tables

**Table 1:** Chemical composition of the tested steels (% by weight).

Steel	C (%)	Mn (%)	Si (%)	P (%)	S (%)	Cr (%)	Ni (%)	Mo (%)	Nb (%)	Cu (%)	Ti (%)	Al (%)
X70SS	0.1	1.04	0.31	0.028	0.008	0.01	0.17	0.01	0.05	0.25	0.027	0.03
X80	0.07	1.84	0.35	0.03	0.008	0.02	0.27	0.01	0.037	0.01	0.018	0.03
X100	0.07	1.96	0.34	0.035	0.007	0.03	0.31	0.18	0.06	0.2	0.02	0.04
30 NiCrMo12	0.3	0.56	0.24	0.035	0.03	0.73	2.69	0.37	0.005	0.02	0	0.03

**Table 2:** Mechanical properties of the tested steels.

Steel	TYS_0.2_^1^ (MPa)	UTS ^2^ (MPa)	YS/UTS	Total Elongation(%)	Charpy Energy at Temperature (J/cm^2^)			
					20 °C	0 °C	−5 °C	−20 °C
X70SS	457	566	0.81	23.5	-	-	270	-
X80	587	659	0.89	20	260	250	-	220
X100	663	750	0.88	19	-	-	344	320
30 NiCrMo12	1097	1135	0.97	8.7	66	64	-	63

^1^ TYS: Yield Tensile Strength at strain at 0.2%; ^2^ UTS: Ultimate Tensile Strength.
